# A single-center study of clinical features of pediatric Sjögren’s syndrome

**DOI:** 10.1186/s12969-023-00902-y

**Published:** 2023-10-13

**Authors:** Ling Hou, Ningning Wang, Chengguang Zhao, Xiuli Wang, Yue Du

**Affiliations:** Department of Pediatrics, Shengjing Hospital of China Medical University, No.36 of Sanhao Street, Heping District, Shenyang, 110004 China

**Keywords:** Autoimmune diseases, Pediatric, Sjögren’s syndrome

## Abstract

**Objective:**

Sjögren’s syndrome (SS) is a rare disease with unclear diagnostic criteria among the children and adolescents. The purpose of this study is to describe the clinical features of pediatric Sjögren’s syndrome and validate with Japanese diagnostic guidelines criteria of 2018.

**Methods:**

We conducted a retrospective analysis of the clinical data of a cohort of 54 patients with pediatric Sjögren’s syndrome admitted to our hospital over a total of 10 years from September 2013 to September 2022.

**Results:**

The ratio of females to males was 49:5 among the 54 children (34 cases of primary SS and 20 cases of secondary SS), the average age of onset of symptoms for the first time was 9.9 years, and the average age at diagnosis was 10.2 years. In terms of subjective symptoms, 7 cases (13.0%) presented with dry mouth and 5 cases (9.3%) reported dry eyes. The positive rates were 9.3% for Schirmer I test, 70.4% for salivary gland function test, and 55.6% for salivary gland ultrasonography. The positive rates were 94.4% for Anti-Ro/SSA antibodies, 66.7% for Anti-La/SSB antibodies, 88.9% for ANA, 59.3% for RF, and the elevation rate of IgG was 63.0%. Among the EULAR Sjögren’s syndrome disease activity index (ESSDAI) domains, the biological, constitutional, glandular, cutaneous, and lymphadenopathy domains were most involved. Treatment consisted of glucocorticoids in 88.9% of the patients in our study and hydroxychloroquine in 92.6%. As per the Japanese version of the clinical practice guidance for Sjögren’s Syndrome in pediatric patients (2018), 5 cases were identified as Definite SS, 35 cases as Probable SS, and 14 cases as Possible SS. With respect to primary and secondary SS, there was essentially no significant difference between the groups in any of the above aspects.

**Conclusions:**

Patients with pediatric SS presented with a wide spectrum of clinical features, a low prevalence of reported symptoms of dry mouth and dry eyes, and various clinical manifestations with multi-system involvement. These are similar to other pediatric study cohorts in terms of epidemiology, auxiliary investigation results, disease activity scores, and treatment. The coincidence between our study and the Japanese version of the clinical practice guidance for Sjögren’s Syndrome in pediatric patients (2018) is good for the diagnosis of pediatric SS.

**Supplementary Information:**

The online version contains supplementary material available at 10.1186/s12969-023-00902-y.

## Introduction

Sjögren’s syndrome (SS) is an autoimmune disease that mainly involves exocrine glands, such as salivary glands and lacrimal glands [1]. It is commonly found in middle-aged females, with a prevalence between 0.01%–0.72% [2]. The clinical features of the disease are highly heterogeneous and can manifest as simple dryness symptoms or as systemic symptoms (characterized by peri-epithelial lymphocytic infiltration of the involved tissues or immune complex deposition) and lymphoma [3].

Pediatric SS is a rare disease with unclear diagnostic criteria and possible underdiagnosis [4]. There are reports of the disease being diagnosed in childhood around the age of 10 years [5], with the common first symptom being salivary gland enlargement [5, 6], and prominent systemic involvement commonly with systemic symptoms (constitutional), lymphadenopathy, glandular lesions, skin lesions, and hematologic lesions [7]. There is no definite diagnostic criteria for pediatric SS [8], and the American-European Consensus Group (AECG) criteria [9] and the American College of Rheumatology (ACR)/European League Against Rheumatism (EULAR) criteria [10] are not applicable to children. While there is no unified diagnostic criteria in China, a recently updated version of diagnostic guidelines for pediatric SS is used in Japan [11], which can be a useful reference.

Paediatric onset of the disease is rarely reported, and poorly defined. In order to better understand and early diagnose of SS in children, we collected data of 54 patients with the clinical diagnosis of pediatric SS admitted to our single center during the last decade, analyzed their clinical features, measured the systemic disease activity index, and compared our data with the Japanese diagnostic guidelines of 2018 [11] for validation of the diagnosis.

## Materials and methods

### Study subjects

In this retrospective study, we collected details of a cohort of 54 pediatric patients with SS with complete clinical data who were admitted to the Department of Pediatric Nephrology and Rheumatology in the Shengjing Hospital of China Medical University (a tertiary center in China) in the past 10 years. All patients who were included were younger than 18 years, and we collected information pertaining to their medical history and relevant laboratory, imaging, and other investigations. Because this is a retrospective, observational, and non-interventional study, ethical review and approval was not required for the study on human participants in accordance with the local legislation and institutional requirements.

### The diagnostic criteria

The diagnostic criteria for pediatric SS which was the opinion of Chinese pediatric rheumatology experts were as follows: After excluding other diseases that could cause dry eyes and/or dry mouth, the following two criteria needed to be satisfied: 1. Presence of objective indicators of dry eyes or salivary gland hypofunction, or significant glandular parenchymal abnormalities that were specific to SS on ultrasonography; 2. Presence of anti-Ro/SSA and/or anti-La/SSB antibodies, or positive labial gland biopsy results, or confirmed systemic rheumatic disease, or positive rheumatoid factor with ANA ≥ 1:320. The Japanese Diagnostic algorithm of Pediatric SS and used criteria (2018) [11] see Supplementary Fig. 1 and Supplementary Table 1.

### Disease activity scores

Disease systemic activity scores were determined using the EULAR SS Disease Activity Index (ESSDAI) scoring system [12].

### Schirmer I test and tear film break-up time

The Schirmer I test was used to assess tear secretion. Wetting of the paper strips was recorded in mm, and a value of ≤ 5 mm was considered to be pathologically reduced tear secretion [11]. Tear film break-up time < 5 s was considered dry eyes [11].

### Salivary gland function test (Salivary gland nuclide imaging)

The 99mTcO4-salivary gland dynamic imaging method was used, and the image acquisition was done for a total of 30 min, and vitamin C stimulation was given at the 20^th^ minute to obtain salivary gland uptake and secretion functions. Impaired uptake and/or secretion functions was considered abnormality.

### Treatment methods

As there is no specific treatment for Sjögren’s Syndrome currently, patients with secondary SS mainly treat the primary disease and primary SS with glucocorticoids (including oral medication, intravenous infusion, and pulse therapy) or immunosuppressive agents according to the condition on diagnosis. The immunosuppressive drugs included hydroxychloroquine, MMF, Belimumab, CTX, and LEF [13].

### Statistical analysis

Statistical analyses were performed using the software IBM SPSS Statistics 26. Continuous variables are described using means and standard deviations, and categorical variables are described as numbers and percentages (%). Two independent samples t-test was used for inter-group comparisons of continuous variables, and chi-square test or Fisher’s exact test was used for inter-group comparisons of categorical variables. Two-tailed tests were used for all significance tests, and a *P* value < 0.05 was considered a statistically significant difference.

## Results

### Sociodemographic characteristics

The cohort in the study consisted of 54 patients, with the ratio of female to male patients being 49:5. The mean age of patients at onset of first symptoms was 9.9 ± 2.6 years (range 2–15 years) and the age at the time of diagnosis was 10.2 ± 2.6 years (range 4–17 years) (Table 1). There were 34 cases of primary SS and 20 cases of secondary SS (19 cases with systemic lupus erythematosus and 1 case with mixed connective tissue disease) in this cohort. The female to male ratio in patients with primary SS was 32:2, the mean age of patients at the time of onset of first symptoms was 9.7 ± 2.8 years, and the age at the time of diagnosis was 10.0 ± 2.7 years. The female to male ratio in patients with secondary SS was 17:3, the mean age of patients at the time of onset of first symptoms was 10.3 ± 2.3 years, and the age at the time of diagnosis was 10.6 ± 2.4 years (Table 1).

**Table 1:** Sociodemographic characteristics and clinical features of pediatric SS patients (*n* = 54)

	No.(*n* = 54)	pSS (*n* = 34)	sSS (*n* = 20)	*P*-value
Age at diagnosis (mean ± SD)	10.2 ± 2.6	10.0 ± 2.706	10.6 ± 2.4	0.416
Age at first sign/symptom (mean ± SD)	9.9 ± 2.6	9.7 ± 2.8	10.3 ± 2.3	0.383
Female: male ratio (%)	49:5(90.7)	32:2(94.1)	17:3(85.0)	0.347^*^
Dry mouth (%)	7(13.0)	2(5.9)	5(25.0)	0.087^*^
Dry eyes (%)	5(9.3)	3(8.8)	2(10.0)	1.000^*^
Fever (%)	29(53.7)	19(55.9)	10(50.0)	0.780^*^
Skin involvement (%)	25(46.3)	14(41.2)	11(55.0)	0.325
Arthralgia (%)	13(24.1)	4(11.8)	9(45.0)	0.009^*^
Arthritis (%)	7(13.0)	2(5.9)	5(25.0)	0.087^*^
Fatigue (%)	6(11.1)	4(11.8)	2(10.0)	1.000^*^
Glandular enlargement (%)	2(3.7)	1(2.9)	1(5.0)	1.000^*^
Peripheral lymphadenopathies (%)	21(38.9)	14(41.2%)	7(35.0)	0.653
Cytopenias (%)	16(29.6)	11(32.4)	5(25.0)	0.568
RTA (%)	5(9.3)	5(14.7)	0(0.0)	0.145^*^
Myalgias (%)	6(11.1)	5(14.7)	1(5.0)	0.395^*^

*SS* Sjögren’s Syndrome, *RTA* Renal Tubular Acidosis

*P*-value: pSS vs sSS

Bold indicates *P* < 0.05

^*^Fisher’s exact test

### Clinical manifestations

Among the 54 pediatric SS patients, the subjective symptoms of dry mouth and dry eyes were 13.0% and 9.3% at diagnosis, 16.6% and 13.0% at whole follow-up, respectively. Half of the patients suffered from fever which could not be explained by infection and malignancy. The remaining symptoms were rash 46.3%, peripheral lymphadenopathies 38.9%, hemocytopenia 29.6%, arthralgia 24.1%, arthritis13.0%, fatigue11.1%, myalgia 11.1%, renal tubular acidosis 9.3%, and salivary gland enlargement 3.7% in order of their incidence (Table 1). Two additional cases started with pulmonary hypertension manifestations (not listed in the table). In terms of the onset of disease as per the primary and secondary SS classification, there was no significant difference in the incidence of clinical manifestations between the two groups, except for the slightly higher incidence of secondary SS in the clinical manifestation of arthralgia (45.0% vs. 11.8%) (Table 1).

### Auxiliary investigations

In the 54 pediatric SS patients in this study, Schirmer I test had a low positive rate (9.3%); the positive rate of salivary gland function test suggesting abnormal salivary gland uptake and/or secretion function was high (70.4%); the rate of abnormal salivary gland ultrasonography suggesting echogenic inhomogeneity, coarse echogenicity, and hypoechogenicity was 55.6%. Just one child did labial biopsy, and showed scattered and focal lymphocyte and plasma cell infiltration around the cathete in primary SS. Laboratory test results showed that the positive rate of Anti-Ro/SSA antibodies was 94.4%, Anti-La/SSB antibodies 66.7% and ANA was positive 88.9%, elevated RF was 59.3%, elevated IgG 63.0%, decreased serum complement C3 was 33.3%, and decreased serum complement C4 was 44.4%. With respect to primary and secondary SS, there was no significant difference between the two groups in the positive rate of the auxiliary investigations except for the slightly higher rate of Anti-Ro/SSA antibodies (100.0% vs. 85.0%) in primary SS (Table 2).

**Table 2:** Auxiliary investigations and laboratory test results of pediatric SS patients (*n* = 54)

	No. (*n* = 54)	pSS (*n* = 34)	sSS (*n* = 20)	*P*-value
Schirmer’s test (%)	5(9.3)	4(11.8)	1(5.0)	0.640^*^
Salivary gland dynamic imaging (%)	38(70.4)	23(67.6)	15(75.0)	0.568
Salivary gland ultrasonography (%)	30(55.6)	21(61.8)	9(45.0)	0.231
Anti-Ro/SSA antibodies-positive (%)	51(94.4)	34(100.0)	17(85.0)	0.046^*^
Anti-La/SSB antibodies-positive (%)	36(66.7)	21(61.8)	15(75.0)	0.319
ANA-positive (%)	48(88.9)	29(85.3)	19(95.0)	0.395^*^
RF-positive (%)	32(59.3)	23(67.6)	9(45.0)	0.102
Low C3 levels (%)	18(33.3)	9(26.5)	9(45.0)	0.163
Low C4 levels (%)	24(44.4)	7(20.6)	17(85.0)	0.000
Elevated IgG (%)	34(63.0)	21(61.8)	13(65.0)	0.812

*SS* Sjögren’s Syndrome, *ANA* antinuclear antibodies, *RF* rheumatoid factor

*P*-value: pSS vs sSS

Bold indicates *P* < 0.05

^*^Fisher’s exact test

### Systemic Disease Activity Index Scores (ESSDAI)

The mean total ESSDAI score at the time of diagnosis for the entire cohort was 4.91 ± 2.71. The main disease activity domains in the ESSDAI score in descending order of frequency were Biological 75.9%, Constitutional 53.7%, Glandular 53.7%, Cutaneous 46.3%, Lymphadenopathy 37.0%, Articular 29.6%, Renal 27.8%, Hematological 27.8%, Muscular 5.6%, CNS 5.6%, PNS 3.7%, and Pulmonary 1.9% (Table 3). Lymph nodes lesions were immune reactive adenopathy (one case with biopsy of cervical lymph node showed lymphoid hyperplasia), no patient in this cohort developed marginal zone prolifgeration or lymphoma. In terms of primary and secondary SS, except for a slightly higher incidence of arthropathy in secondary SS (50.0% vs. 17.6%), there was no significant difference between the two groups in the incidence of other domains (Table 3).

**Table 3:** Results of the Systemic Disease Activity score (ESSDAI) of pediatric SS patients (*n* = 54)

	No. (*n* = 54)	pSS (*n* = 34)	sSS (*n* = 20)	*P*-value
ESSDAI score (mean ± SD)	4.91 ± 2.71	4.53 ± 2.44	5.55 ± 3.07	0.184
ESSDAI domains (score ≥ 1)				
Constitutional (%)	29(53.7)	19(55.9)	10(50.0)	0.675
Lymphadenopathy (%)	20(37.0)	13(38.2)	7(35.0)	0.812
Glandular (%)	4(53.7)	2(5.9)	2(10.0)	0.622^*^
Articular (%)	16(29.6)	6(17.6)	10(50.0)	**0.012**
Cutaneous (%)	25(46.3)	14(41.2)	11(55.0)	0.325
Pulmonary (%)	1(1.9)	0(0.0)	1(5.0)	0.370^*^
Renal (%)	15(27.8)	9(26.5)	6(30.0)	0.780
Muscular (%)	3(5.6)	3(8.8)	0(0.0)	0.287^*^
PNS (%)	2(3.7)	1(2.9)	1(5.0)	1.000^*^
CNS (%)	3(5.6)	1(2.9)	2(10.0)	0.548^*^
Haematological (%)	15(27.8)	10(29.4)	5(25.0)	0.727
Biological (%)	41(75.9)	24(70.6)	17(85.0)	0.329^*^

*SS* Sjögren’s Syndrome, *ESSDAI* EULAR Sjögren’s syndrome disease activity index, *PNS* peripheral nervous system, *CNS* Central nervous system

*P*-value: pSS vs sSS

Bold indicates *P* < 0.05

^*^Fisher’s exact test

### Comparison of ESSDAI domain score distribution

The comparison of the main symptoms, serologic tests, and ESSDAI domains between this study cohort and six other pediatric SS cohorts is presented in Table 4, which shows that the incidence of subjective dry mouth and dry eyes symptoms of the patients in this cohort was significantly lower than those reported in the others [5, 7, 14], but similar to these reports [15–17]. Laboratory investigations showed that the proportions of positive ANA, elevated RF, positive Anti-Ro/SSA antibodies, and positive Anti-La/SSB antibodies were not significantly different (Table 4). In terms of ESSDAI domains, the top five sites in this cohort were serologic changes, systemic symptoms, glandular lesions, skin lesions, and lymph node lesions, the results of the comparison between this cohort and the other four cohorts were basically consistent (Table 4).

**Table 4:** Clinical and laboratory features of pediatric SS patients

Author (the number of patients)	Present study (*n* = 54)	Gong et al. [16] (*n* = 39)	Liu et al. [17] (*n* = 49)	Ramos-Casals et al. [7] (*n* = 158)	Legger et al. [14] (*n* = 23)	Kobayashi et al. [15] (*n* = 25)	Cimaz et al. [5] (*n* = 40)
Data of country	China (Liaoning)	China (Shanghai)	China (Shanghai)	Multi-center	Netherlands	Japan (Hokkaido)	Multi-center
Reported year	-	2023	2023	2021	2021	2019	2003
Sicca symptoms	Oral 13.0% Ocular 9.3%	Oral 17.9% Ocular 10.4%	Oral 18.4% Ocular 18.4%	Oral 79.7% Ocular 70.3%	Oral 52.2% Ocular 26.1%	16%	35%
ANA	88.9%	94.9%	93.9%	90.3%	95.7%	92%	85.0%
RF	59.3%	43.6%	35.6%	67.6%	82.6%	76%	75.0%
Anti-Ro/SSA antibodies	94.4%	100%	85.7%	82.7%	82.6%	80%	73.6%
Anti-La/SSB antibodies	66.7%	46.1%	32.7%	61.9%	56.5%	64%	
ESSDAI domains							
Constitutional	53.7%	28.2%		21.9%	34.8%	68%	10%
Lymphadenopathy	37.0%	23.1%		25.2%	17.4%	8%	(7.5%)
Glandular	53.7%	20.5%		47.1%	69.6%	44%	72.5% (57.5%)
Articular	29.6%	10.3%		26.5%	27.1%	44%	17.5%
Cutaneous	46.3%	35.9%		12.3%	8.7%	28%	
Pulmonary	1.9%	7.7%		5.2%	4.3%	0%	
Renal	27.8%	12.8%		4.5%	0%	16%	(8.6%)
Muscular	5.6%	0%		1.9%	0%	4%	
PNS	3.7%	2.6%		0%	0%	4%	
CNS	5.6%	0%		0.6%	0%	12%	(5%)
Haematological	27.8%	28.2%		28.4%	8.7%	4%	
Biological	75.9%	79.5%		54.2%	47.8%	96%	53.1%

*ANA* antinuclear antibodies, *RF* rheumatoid factor, *ESSDAI* EULAR Sjögren’s syndrome disease activity index, *PNS* peripheral nervous system, *CNS* Central nervous system

### Treatment details

In this study cohort, 88.9% patients were treated with glucocorticoids and 92.6% patients were treated with hydroxychloroquine. In terms of immunosuppressive agents, 24.1% were treated with MMF, 7.4% with Belimumab, 5.6% with CTX, and 1.9% with LEF. In primary SS group, all patients were treated with glucocorticoids and hydroxychloroquine. In terms of immunosuppressive agents, 17.6% were treated with MMF, 5.9% with Belimumab, 2.9% with CTX, and 2.9% with LEF. In terms of primary and secondary SS, there were no significant differences between the two groups in terms of the treatment received (Supplementary Table 2). The followed-up time was 1 to 6 years. One with fever and rash relapse, one with thrombocytopenia relaspe, one with new arthritis, and one with new RTA, dry mouth and dry eyes, neuromyelitis optica and hemiplegia in primary SS. One with dry mouth, one with dry eye and two with seizure in secondary SS (Supplementary Table 3).

### Validation with Japanese diagnostic guidelines criteria of 2018

According to the Japanese Diagnostic Guidelines for Pediatric Sjögren’s Syndrome (2018) [11], in the 54 pediatric SS patients, 5 cases were identified as Definite SS, 35 cases as Probable SS, 14 cases as Possible SS, and no case was classified as Needs follow-up and Possibly non-SS, which suggested that the cohort had a high rate of compliance with this diagnostic guideline. There was no significant difference between the primary and secondary SS groups in terms of validation of diagnostic criteria (Supplementary Table 2). Out of the total 54 patients in our cohort, 7.4%(4 cases) fulfilled the AECG criteria. Out of the 34 pSS patients in our cohort, 8.8%(3 cases), 14.7% (5 cases)and 52.9%(18 cases) fulfilled the AECG criteria, ACR/EULAR criteria and proposed juvenile pSS criteria, respectively (Supplementary Table 2).

## Discussion

Although the onset of SS is mainly in middle-age, epidemiological studies have shown that the disease can occur at any age and has been diagnosed in patients aged 2 [18] to 97 years [19]. The data available so far indicates that SS is a very rare disease in children [20]. The number of studies and reported cases of pediatric-onset SS are very limited compared to that of adult-onset SS. The 54 patients included in this cohort were around 10 years of age at the time of diagnosis, which was generally consistent with the findings of other pediatric cohort studies, and in terms of gender composition, our cohort had more girls and the female to male ratio in our cohort was 49:5 (9.8:1), when compared to the ratio of around 6:1 reported in other cohorts [5–8, 14–17].

In addition to dryness symptoms, patients with SS can have extensive extraglandular multiorgan and multisystem involvement, and in some cases, the organ damage can precede the dryness symptoms. Skin involvement is more common and includes purpura-like rash, erythema nodosum, and Raynaud’s phenomenon [21]. In our cohort, the skin involvement was also common being present in 46.3%. Involvement of the skeletal muscular system may be present with joint swelling and pain, arthritis, myalgia [21]. In our cohort, the skeletal muscular system involvement was 11.1%. Renal involvement mainly involves the distal renal tubules, and renal tubular acidosis is more common in pediatric SS patients [21]. In our cohort, renal tubular acidosis was 9.3%. In the hematological system, anemia, leukopenia, thrombocytopenia, and hyperimmunoglobulinemia is one of the features of SS [19]. In our cohort, cytopenias was 29.6%, and hyperimmunoglobulinemia was 63.0%. The incidence of lymphoma is significantly higher in patients with SS compared to the normal population. But lymphoma was not found in our cohort. The respiratory tract is most dominated by interstitial lung disease [21]. Involvement of exocrine glands in the mucosal layer of the digestive tract may result in atrophic gastritis, gastroesophageal reflux, dyspepsia [21]. Neurological involvement is less frequent and mostly manifests as impairment of peripheral nerve functions, and it can also coexist with neuromyelitis optica spectrum disorders [21]. In our cohort, the neurological involvement was 5.6%.

In this cohort, the pediatric dry symptoms were uncommon, and few patients presented with parotid enlargement, but more patients visited the hospital with extra-glandular symptoms manifesting mostly as fever, joint symptoms, lymph node lesions, and rare conditions such as renal tubular acidosis and pulmonary hypertension. This could be due to the fact that the build-up of exocrine gland dysfunction takes time, and it did not progress to visible symptoms in pediatric patients due to their young age. In contrast to our findings, Ramos-Casals et al. [7] reported a higher proportion of dryness symptoms in their cohort (*n* = 158), with 79.7% reporting dry mouth and 70.3% reporting dry eyes, and this may be related to the different inclusion criteria of cases in the cohorts. Inclusion in their cohort was based on the 2002 AECG and/or 2016 ACR/ EULAR classification criteria, where the premise of dry eye and dry mouth symptoms were the basis for inclusion, and hence, the difference in the incidence of subjective symptoms of dry mouth and dry eyes was more pronounced and may be due to case selection bias [7]. However, in Asian cohort studies, symptoms of dryness were generally lower [15–17], suggesting that there may also be racial differences. This could similarly explain the large variation in the proportion of subjective symptoms of dry mouth and dry eyes (16%–52.2%) reported in several other pediatric cohorts [5–7, 14–17]

Cimaz R [5] and Stiller M [6] reported recurrent parotid swelling was the most common clinical feature being present in 57.5% and 47.8%, respectively. But in our cohort, it was only 3.7%. The reason for the difference is not clear, it may be related to the different ethnic composition of the cohort.

The examination of the exocrine glands consists mainly of the salivary gland and lacrimal gland examinations [22]. Salivary secretion function can be evaluated by measuring salivary flow rate, including post-stimulation flow rate (e.g., chewing gum test, Saxon test) or resting/non-stimulation flow rate. Parotid gland imaging or 99mTcO4-nuclein imaging are also commonly used for detection. Salivary gland biopsy, which has low acceptance in pediatric patients, can visualize glandular involvement, and focal infiltration of lymphoid cells can be seen around the glandular ducts in patients with SS. Salivary gland ultrasonography and magnetic resonance imaging are also used [8]. In our center, nuclide imaging is often used to evaluate the secretory and excretory functions of the salivary gland and ultrasonography is utilized to observe the parenchymal damage of the gland. In this cohort, the positive rate was 70.4% for nuclide imaging and 55.6% for ultrasonography. Although the clinical manifestations of glandular enlargement were few, the ratio of glandular function and parenchymal damage was actually high, which suggested that there was mostly objective evidence in the pediatric salivary gland examination, but the external manifestations were absent. The means commonly used for lacrimal gland examination include Schirmer’s test, tear break-up time, and corneal and conjunctival staining score. The Schirmer I test, a method that does not use surface anesthesia, is used to determine the secretory function of the primary lacrimal gland and is generally well tolerated by children. The positive rate for Schirmer I test was not high in patients in our cohort, which is consistent with the low incidence of subjective dry eye symptoms of patients, and our results indicated that in pediatric SS, the involvement of salivary glands was greater than that of lacrimal glands.

In terms of laboratory investigations, the positive rate of Anti-Ro/SSA antibodies in our cohort (94.4%) was slightly higher than what others reported (73.6%–82.7%), and the positive rates of other indicators such as Anti-La/SSB antibodies, ANA, RF, and IgG were not significantly different [5–8, 14–17], and the variation in these indicators was consistent with the features of SS. The presence of autoantibodies is of considerable value in the diagnosis of SS, and studies have shown that serologic evidence is present approximately 18–20 years before the diagnosis is confirmed [23], therefore, it is also highly suggestive in the diagnosis of pediatric SS.

Pediatric SS is undeniably a systemic disease (90% of cases have systemic activity at the time of diagnosis, defined as ESSDAI score ≥ 1). Age at diagnosis is a critical deciding factor for systemic disease expression in primary SS, and an international cohort study showed that the highest ESSDAI score was in the 18–35 years group [19]. The ESSDAI score of our cohort was 4.91 ± 2.71, which is slightly lower than that reported by other cohorts [5–8, 14–17]. The top five sites in this cohort were serologic changes, systemic symptoms, glandular lesions, skin lesions, and lymph node lesions; The top five areas reported by Ramos-Casals et al. [7] (*n* = 158) were serologic changes, glandular lesions, hematologic lesions, lymph node lesions, and arthropathy; the top five areas reported by Legger et al. [14] (*n* = 23) were glandular lesions, serological changes, systemic symptoms, arthropathy, and lymph node lesions; those reported by Kobayashi et al. [15] (*n* = 25) were serological changes, systemic symptoms, glandular lesions, arthropathy, and skin lesions; and those reported by Cimaz et al. [5] (*n* = 40) were glandular lesions, arthropathy, systemic symptoms, renal lesions, and lymph node lesions, and those reported by Gong Y et al. [16] (*n* = 39) were serologic changes, skin lesions, systemic symptoms, hematologic lesions, and lymph node lesions, and there was no significant difference in the domains that were frequently involved [5–8, 14–16].

There is no specific treatment for SS that can reverse impaired glandular function and cure the disease, and patients are usually treated symptomatically to improve their quality of life. The vast majority of patients in our cohort had been treated with glucocorticoid and hydroxychloroquine, which were mainly administered to treat the extra-glandular symptoms, control the patient’s systemic inflammatory response, and protect the gland from further damage. Immunosuppressive agents were used in a low proportion, while MMF was commonly used. The treatment was also basically consistent with the reports of pediatric SS [8].

Starting with the Bloch criteria in 1965 [24], there are more than a dozen diagnostic and classification criteria for SS to date, but all are based on data from adult cohorts and have not been validated in pediatric cohorts. In adult SS, the 2002 AECG and/or 2016 ACR/ EULAR classification criteria are often used, but in pediatric SS, the compliance of these two criteria in clinical use is not high [8, 25]. 1999 proposed juvenile pSS criteria [26] have not been widely verified, and no formal diagnosis and classification criteria for children’s SS have been proposed yet. Our pSS patients showed only 8.8%, 14.7% and 52.9% met AECG criteria and ACR/EULAR criteria and proposed juvenile pSS criteria, respectively. New classification criteria was proposed in Japan in 2015 with the aim to identify pediatric SS cases at an early stage [27]. The criteria were designed to have separate scores for serology (S-score) and glandular involvement (G-score), and the score items took into account the special features of children, such as the difficulty of performing labial gland biopsy in pediatric patients, which was assigned fewer points, and further emphasized the importance of autoantibodies for the diagnosis of SS, and dryness symptoms were only one of the manifestations that needed clinical attention. The classification categories include definite SS, probable SS, possible SS, needs follow-up, and possibly non-SS [11]. An official diagnostic guideline for these criteria was published in 2018 [11] and the same set of criteria is used for primary SS and secondary SS. This study did not adopt the current international standards [9, 10], but based on the opinion of Chinese pediatric rheumatology experts, focusing on substantive evidence of exocrine glands involvement rather than sicca symptoms, combined with serological characteristics, aimed at identifying the clinical characteristics of this group disease in early stage for early diagnosis and treatment. Our pediatric cohort showed good compliance with the Japanese guideline, with 9.3% identified as Definite SS, 61.8% as Probable SS, and 26.5% as Possible SS. Taking into account the above discussion on the sicca symptoms, the incidence in Asian cohort is more similar (Table 4) [15–17], suggesting that the reason why the Japanese guideline is more applicable to our cohort may also be related to race.

The characteristics of SS such as photosensitive rash, arthritis, positive autoantibodies may also be observed in the other autoimmune diseases (including rheumatic arthritis and systemic lupus erythematosus (SLE). 8.3% -19.0% of SLE patients accompanied with SS, even some cases develop SLE several years after being diagnosed with SS [28–30]. Researches on adults found that, SLE with SS appears to constitute a subgroup of patients with distinct clinical, serologic, pathologic, and immunogenetic features, in whom SS is expressed as an overlapping entity and is largely similar to primary SS [30]. From this cohort study, we also found that there was no significant difference in clinical manifestations between primary and secondary SS, and sicca symptoms in children were uncommon, which made the diagnosie of SS face great challenge in distinguishing primary and secondary. Fever is atypical symptom, and in the absence of infections and tumors, which only indicates systemic inflammation and has no specificity for diagnosis. In addition, in our subgroup analysis, there were no significant differences between primary and secondary SS with regard to epidemiological features, clinical manifestations, auxiliary investigations, disease activity scores, and treatment, except for individual aspects, which suggested that these patients had a common pathogenesis that may be related to the similar autoantibody spectrum. However, from another perspective, since primary and secondary SS have such similarities in many clinical aspects, it is not so urgent to distinguish them early. Only closely follow-up, observation, and treatment are needed.

As is common among rheumatic diseases, the lack of an objective standard for diagnosis of SS in children requires the use of expert opinion as our gold standard for inclusion. This also leads to the evaluation of children being performed in a non-standardized fashion. Children with mild dryness may not seek medical attention resulting in a lower perceived prevalence of dryness and possibly the disease. And this was a retrospective clinical study, and the limitation is that the small sample size may have led to some bias in our findings. A larger sample size is needed in the future for more in-depth enquiry.

In conclusion, based on the analysis of clinical cases of pediatric SS in this study, we found that the incidence of the subjective symptom dry mouth and dry eyes was low and that children were more likely to develop various clinical manifestations of multisystem involvement. The findings of this study are similar to other pediatric study cohorts in terms of epidemiology, auxiliary investigation results, disease activity scores, and treatment. The Japanese Diagnostic Guidelines for Pediatric Sjögren’s Syndrome (2018) may be more suitable for the diagnosis of pediatric Sjögren’s syndrome in china.

### Supplementary Information

**Additional file 1: Supplementary Figure 1.** The Japanese diagnostic algorithm of Sjögren’s Syndrome in children and adolescents (2018).

**Additional file 2: Supplementary Table 1.** The Japanese diagnostic criteria of Sjögren’s Syndrome in children and adolescents (2018). **Supplementary Table 2.** Treatment of pediatric SS patients and classification results based on the 2018 Japanese diagnostic guidelines (*n* = 54). **Supplementary Table 3.** Symptoms at diagnosis and during disease evolution.

## Data Availability

All data generated or analysed during this study are included in this article. Further enquiries can be directed to the corresponding author.

## Abbreviations

ANA: Antinuclear antibodies
RF: Rheumatoid factor
SS: Sjögren’s syndrome
AECG: American-European Consensus Group
ACR: American College of Rheumatology
EULAR: European League Against Rheumatism
ESSDAI: EULAR Sjögren’s syndrome disease activity index
RTA: Renal Tubular Acidosis
PNS: Peripheral nervous system
CNS: Central nervous system
LEF: Leflunomide
CTX: Cyclophosphamide
MMF: Mycophenolate mofeti
