# Supplementary figures and images for: A single-center study of clinical features of pediatric Sjögren’s syndrome
Source: Pediatr Rheumatol Online J. 2023 Oct 13;21:119. doi: 10.1186/s12969-023-00902-y (PMC10571342; doi:10.1186/s12969-023-00902-y)

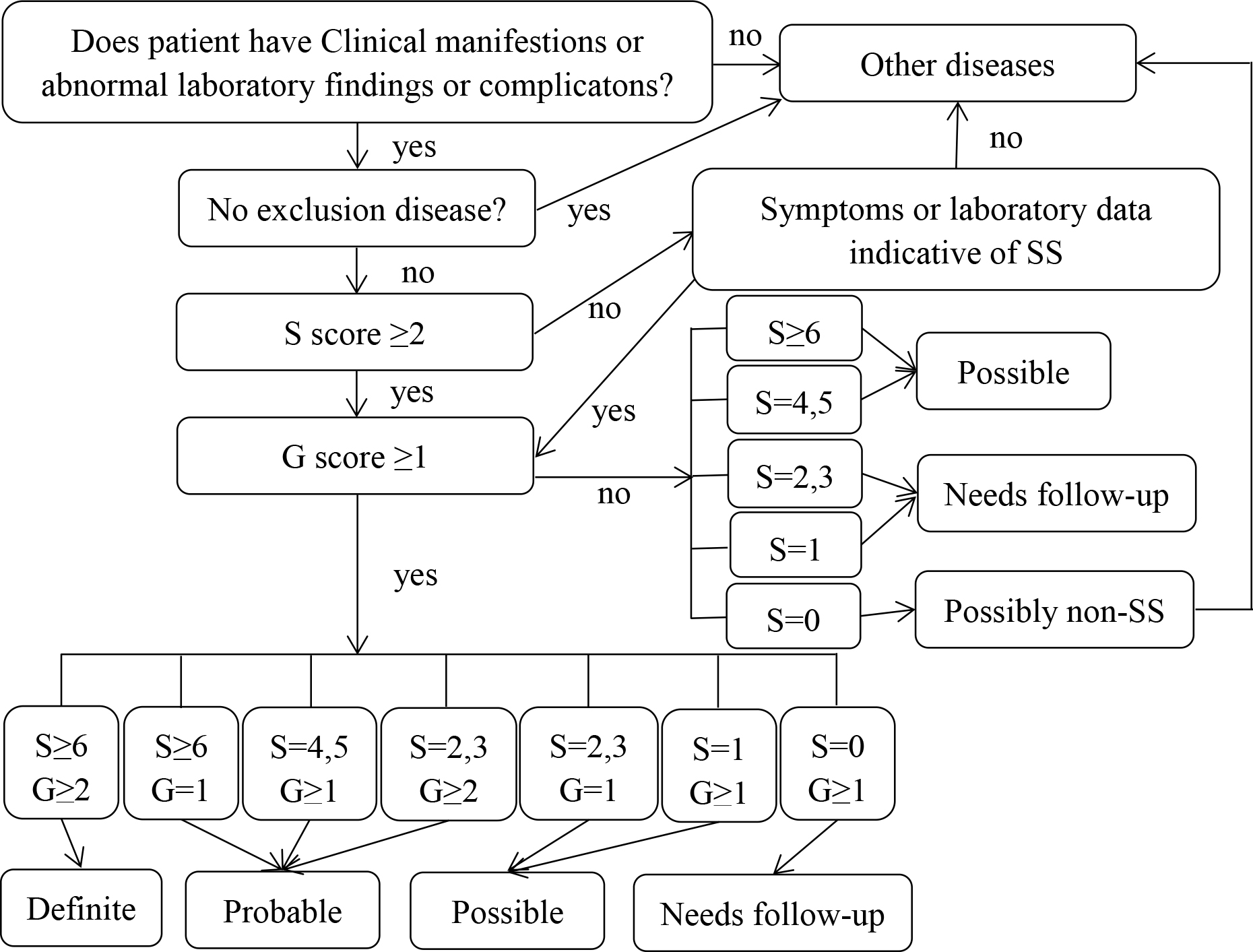

Supplement: Supplementary file 1 — Additional file 1: Supplementary Figure 1. The Japanese diagnostic algorithm of Sjögren’s Syndrome in children and adolescents (2018). [file 12969_2023_902_MOESM1_ESM.jpg]
