# Supplementary material for: A single-center study of clinical features of pediatric Sjögren’s syndrome
Source: Pediatr Rheumatol Online J. 2023 Oct 13;21:119. doi: 10.1186/s12969-023-00902-y (PMC10571342; doi:10.1186/s12969-023-00902-y)
Supplement: Supplementary file 2 — Additional file 2: Supplementary Table 1. The Japanese diagnostic criteria of Sjögren’s Syndrome in children and adolescents (2018). Supplementary Table 2. Treatment of pediatric SS patients and classification results based on the 2018 Japanese diagnostic guidelines (n = 54). Supplementary Table 3. Symptoms at diagnosis and during disease evolution. [file 12969_2023_902_MOESM2_ESM.docx]

Supplementary table 1 The Japanese diagnostic criteria of Sjögren’s Syndrome in children and adolescents (2018)

| S Score | Criteria | Score |
| --- | --- | --- |
| IgG level | Higher than 97.5 percentile in the corresponding age group | 1 |
| Anti-nuclear antibody | 1:40-1:80 | 1 |
|  | 1:160 | 2 |
|  | ≥1:320 | 3 |
| Rheumatoid factor | ≥15.0 U/L | 3 |
| Either anti-SS-A/Ro  antibody or anti-SS-B/La  antibody | Result from the Ouchterlony method 1:1, higher than reference value by ELISA | 6 |
| G Score | Criteria | Score |
| Labial minor salivary gland biopsy | Cellular infiltration is apparent but focus (periductal infiltrate  of more than 50 mononuclear cells)  < 1 focus / 4 mm^2^ | 1 |
|  | > 1 focus / 4 mm^2^ | 2 |
| Parotid gland sialography | Stage in the Rubin-Holt Classification ≥1 | 2 |
| Salivary gland scintigraphy | Reduction of uptake or secretion in any of the 4 major salivary glands | 1 |
| Salivary secretion | Result of Saxon test 2.0 g / 2 minutes  or  Production rate of resting saliva 1.5 mL / 15 minutes  or  Result of chewing gum test 10 mL / 10 minutes | 1 |
| Lachrymal gland | Both Schirmer’s test  ≤5 mm / 5 minutes and a positive rose bengal test (van Bijsterveld score ≥3) | 2 |
|  | Both Schirmer’s test  ≤5 mm / 5 minutes and positive fluorescein staining | 2 |
|  | ACR score (staining of cornea and conjunctiva)≥3 | 2 |

1.1 The criteria of S Score and G Score

1.2 The diagnostics guidance.

| S Score | G Score | | |
| --- | --- | --- | --- |
|  | ≥2 | 1 | 0 |
| ≥6 | Definite | probable | possible |
| 5 | Probable | Probable | possible |
| 4 | Probable | Probable | possible |
| 3 | Probable | possible | needs follow-up |
| 2 | Probable | possible | needs follow-up |
| 1 | possible | possible | needs follow-up |
| 0 | needs follow-up | needs follow-up | possibly non-SS |

Supplementary table 2 Treatment of pediatric SS patients and classification results based on the 2018 Japanese diagnostic guidelines (n = 54)

|  | No.(n=54) | pSS (n=34) | sSS (n=20) | *P*-value |
| --- | --- | --- | --- | --- |
| Steroid(%) | 48(88.9) | 30(88.2) | 18(90.0) | 1.000* |
| Hydroxychloroquine(%) | 50(92.6) | 30(88.2) | 20(100.0) | 0.285* |
| MMF(%) | 13(24.1) | 6(17.6) | 7(35.0) | 0.194* |
| Belimumab(%) | 4(7.4) | 2(5.9) | 2(10.0) | 0.622* |
| CTX(%) | 3(5.6) | 1(2.9) | 2(10.0) | 0.548* |
| LEF(%) | 1(1.9) | 1(2.9) | 0(0.0) | 1.000* |
| Japanese criteria(2018) |  |  |  |  |
| Definite SS(%) | 5(9.3) | 4(11.8) | 1(5.0) | 0.640* |
| Probable SS(%) | 35(61.8) | 21(70.0) | 14(64.8) | 0.541 |
| Possible SS(%) | 14(26.5) | 9(25.0) | 5(25.9) | 0.905 |
| Needs follow-up(%) | 0(0) | 0(0) | 0(0) | - |
| Possibly non-SS(%) | 0(0) | 0(0) | 0(0) | - |
| AECG criteria(%) | 4(7.4) | 3(8.8) | 1(5.0) | 1.000* |
| ACR/EULAR(%) |  | 5(14.7) |  |  |
| Proposal juvenile pSS criteria(1999)(%) |  | 18(52.9) |  |  |

*Fisher's exact test

SS: Sjögren’s Syndrome, MMF: Mycophenolate mofeti, CTX: Cyclophosphamide, LEF: Leflunomide

*P*-value: pSS vs sSS

Supplementary table 3 Symptoms at diagnosis and during disease evolution

| Symptoms | at diagnosis (No.) | | during disease evolution(No.) | |
| --- | --- | --- | --- | --- |
|  | pSS(34) | sSS(20) | pSS(34) | sSS(20) |
| Dry mouth | 2 | 5 | 1 | 1 |
| Dry eyes | 3 | 2 | 1 | 1 |
| Fever | 19 | 10 | 0 | 0 |
| Skin involvement | 14 | 11 | 1 | 0 |
| Arthralgia | 4 | 9 | 0 | 0 |
| Arthritis | 2 | 5 | 1 | 0 |
| Fatigue | 4 | 2 | 0 | 0 |
| Glandular enlargement | 1 | 1 | 0 | 0 |
| Peripheral lymphadenopathies | 14 | 7 | 0 | 0 |
| Cytopenias | 11 | 5 | 1 | 0 |
| RTA | 5 | 0 | 1 | 0 |
| Myalgias | 5 | 1 | 0 | 0 |
| Seizure | 0 | 0 | 0 | 2 |
| Neuromyelitis optica | 0 | 0 | 1 | 0 |
| Hemiplegia | 0 | 0 | 1 | 0 |
